# Family history of cancer, personal history of medical conditions and risk of oral cavity cancer in France: the ICARE study

**DOI:** 10.1186/1471-2407-13-560

**Published:** 2013-11-28

**Authors:** Loredana Radoï, Sophie Paget-Bailly, Florence Guida, Diane Cyr, Gwenn Menvielle, Annie Schmaus, Matthieu Carton, Sylvie Cénée, Marie Sanchez, Anne-Valérie Guizard, Brigitte Trétarre, Isabelle Stücker, Danièle Luce

**Affiliations:** 1 Centre for Research in Epidemiology and Population Health (CESP), Inserm U1018, Epidemiology of Occupational and Social Determinants of Health Team, F-94807 Villejuif, France; 2 University Versailles St-Quentin, F-78035 Versailles, France; 3 Centre for research in Epidemiology and Population Health (CESP), Inserm U1018, Environmental Epidemiology of Cancer Team, F-94807 Villejuif, France; 4 University Paris-Sud, UMRS 1018, F-94807 Villejuif, France; 5 Calvados Cancer Registry, F-1400 Caen, France; 6 Hérault Cancer Registry, F-34298 Montpellier, France; 7 Inserm U1085, Irset, Faculté de Médecine, Campus de Fouillole, BP 145, 97154 Pointe-à-Pitre, Guadeloupe French West Indies

**Keywords:** Family history, Medical conditions, Oral cavity cancer, Risk factors, Case–control study

## Abstract

**Background:**

The aim of this study was to evaluate the role of family history of cancer and personal history of other medical conditions in the aetiology of the oral cavity cancer in France.

**Methods:**

We used data from 689 cases of oral cavity squamous cell carcinoma and 3481 controls included in a population-based case–control study, the ICARE study. Odds-ratios (ORs) associated with family history of cancer and personal medical conditions and their 95% confidence intervals (95% CI) were estimated by unconditional logistic regression and were adjusted for age, gender, area of residence, education, body mass index, tobacco smoking and alcohol drinking.

**Results:**

Personal history of oral candidiasis was related to a significantly increased risk of oral cavity cancer (OR 5.0, 95% CI 2.1-12.1). History of head and neck cancers among the first-degree relatives was associated with an OR of 1.9 (95% CI 1.2-2.8). The risk increased with the number of first-degree relatives with head and neck cancer.

**Conclusion:**

A family history of head and neck cancer is a marker of an increased risk of oral cavity cancer and should be taken into account to target prevention efforts and screening. Further studies are needed to clarify the association between oral cavity cancer and personal history of candidiasis.

## Background

Oral cavity cancer (International Classification of Diseases 10th revision (ICD-10) codes C00-C08 [1]) is an important public health burden with an annual worldwide incidence estimated at approximately 263,000 cases, and mortality at 127,000 [2]. Among developed countries, France has the highest age-standardized incidence rate for males (7.6/100,000) and one of the highest for females (1.5/100,000) [3]. As is the case for the other sites of upper aerodigestive tract (UADT), tobacco and alcohol consumption are the main risk factors for oral cavity cancer [4,5].

Besides the role of human papilloma viruses (HPV) 16 and 18 in the aetiology of UADT cancers, few other conditions such as herpetic infection [6-11], candidiasis [6,10,11], warts [6,9-11], and gastro-oesophageal reflux [7] have been investigated. The results of the epidemiological studies on the role of these medical conditions in the occurrence of UADT cancers are contradictory and the underlying mechanisms are not complete elucidated.

Other risk factors, such as genetic polymorphism in genes involved in the metabolism of tobacco and alcohol carcinogens and DNA repair seems to play a role in the development of UADT cancers [12-19]. Few epidemiological studies considered the risk of UADT cancers in relatives of subjects with cancer history [20-26]. Familial clustering of UADT cancers may indicate that genetic factors play a role in the process of carcinogenesis, but may also reflect a tendency of relatives to have similar behaviour towards tobacco and alcohol. Limited data are available on the combined effect of family history, and tobacco and alcohol consumption [20,23,26]. The literature is contrasted about whether the cancer risk varies according to UADT subsite, gender, type of affected relative (parents, siblings), and their cancer site.

The present work aimed to investigate the role of family history of cancer and personal medical history in the aetiology of oral cavity cancer in France using data from a large case–control study, the ICARE study.

## Methods

### ICARE study

The ICARE study (Investigation of occupational and environmental CAuses of REspiratory cancers) is a multicentre population-based case–control study on lung and upper aerodigestive tract cancers carried out from 2001 to 2007 in 10 French administrative areas (“départements”) covered by a general cancer registry. This study was set up to explore the role of lifestyle, environmental and occupational risk factors in lung and UADT cancers. The study design has been described in details elsewhere [27].

Briefly, all newly diagnosed primary oral cavity, pharynx, larynx, sinusal cavities, trachea and lung cancers were selected. Only histologically confirmed cases aged 75 or younger at interview, identified between 2001 and 2007, and residing in one of the 10 départements, were eligible. Clinical and anatomo-pathology reports were reviewed to determine topography and histological type of the tumours according to the International Classification of Diseases for Oncology [28]. All histological types were included.

Controls were selected from the general population by random digit dialling [29]. The controls were frequency-matched to the cases by age, gender and area of residence (“département”). Additional stratification ensured that controls were representative of the population of the “département” in terms of socio-economic status based on the last job held.

### Present analysis

The present analysis included all ICARE controls and only the cases with oral cavity cancer (ICD-10 codes C01-C06).

Among the 1316 oral cavity cancer cases identified as eligible, 196 could not be reached, 81 were deceased and 71 were too sick to be interviewed. Of the 968 cases who were contacted, 176 refused to participate and 792 (81.8%) answered the questionnaire. We focused only on the cases with squamous cell carcinoma (772 subjects, 97.5% of all cases with oral cavity cancer).

Of 4673 eligible controls, 4411 were contacted, and 3555 (80.6%) agreed to participate.

### Data collection

Trained interviewers administered a detailed standardised questionnaire during face-to-face interviews. If the subject was too sick to be interviewed, a shortened version of the questionnaire was used to interview him or a next-of-kin. Among the 772 subjects with squamous cell carcinoma of the oral cavity, 689 (89.2%) filled a complete questionnaire and 83 (10.8%) a shortened questionnaire. Among controls, 3481 (97.9%) filled a complete questionnaire and 74 a shortened questionnaire (2.1%). As the shortened version of the questionnaire did not contain information about family history of cancer and medical conditions, the present analysis was based on 689 cases with squamous cell carcinoma and 3481 controls, all with a complete questionnaire.

The complete questionnaire consisted of the following items: socio-demographic characteristics (age, gender, birth country, education level, marital status), residential history, personal medical history, family history of cancer, detailed tobacco and alcohol consumption (quantity, duration, type of product, age at starting, time since cessation), non-alcoholic beverage consumption (coffee, tea), anthropometric variables (height, weight at interview, two years before and at age 30), detailed lifelong job history and occupational exposures.

To ascertain personal medical history, study participants were asked if, throughout their lives, they had ever had (“yes, no, or don’t know”) any of the following diseases: tuberculosis, chronic bronchitis, asthma, recurrent rhinitis, nasal polyps, recurrent nose bleeds, recurrent sinusitis, gastro-oesophageal reflux (heartburn or regurgitation), herpes, candidiasis, and warts. If the answer was “yes”, the subjects were asked to specify the age at first occurrence, the treatment and if the diagnosis was made by a doctor. Subjects reporting having ever had herpes, candidiasis or warts were asked to specify the location: lip and genitals for herpes, oral cavity and genitals for candidiasis, and hands, feet and head and neck for warts.

To ascertain the family history of cancer, subjects were first asked to give the year of birth of their biological mother and father, and of their full brothers or sisters (“brother or sister having the same mother and the same father than you”). Then, they were asked for each of these relatives if she/he had ever had a cancer (“yes, no or don’t know”). If the answer was “yes”, the subjects were asked to specify the age at cancer diagnosis and if possible the type of cancer. No verification of the cancer diagnosis in the relatives was performed.

### Statistical analysis

We used unconditional logistic regression models to calculate odds ratios (OR) and their 95% confidence intervals (95% CI). All *p-*values were derived from two-sided statistical tests.

All logistic regression models controlled for age (≤ 50, 51–59, 60–69, ≥ 70 years), gender, area of residence, education level (primary or less, vocational secondary, general secondary and university), BMI two years before the interview (categorical, according to the classification of the World Health Organization [30]: < 18.5, 18.5-24.9, 25.0-29.9, ≥ 30 kg/m^2^). Previous analyses of our data showed that the variables that best characterize the association between tobacco and alcohol consumption and oral cancer risk were smoking status, smoking duration, daily quantity of tobacco smoked and daily quantity of alcohol drinking [31]. To control for smoking, we used smoking status (never, current, former), average daily quantity of tobacco smoked (1–19, 20–39, ≥ 40 grams), and duration of smoking (1–30, 31–40, > 40 years) [32]. Average daily quantity of alcohol drinking in quartiles (never, < 0.6, 0.6-2.0, 2.1-4.5, > 4.5 standard glasses) was included in the models to adjust for alcohol drinking. The quantity of pure alcohol contained in a standard glass (15 cl of wine, 30 cl of beer, 5 cl of spirits, 10 cl of aperitif, and 30 cl of cider) is the same for each type of alcoholic beverage.

We analysed the risk of oral cavity cancer related to the personal medical history using two variables: all medical conditions self-reported by the subjects and medical conditions reportedly diagnosed by a doctor. The date of interview was used as the date of reference for both cases and controls. This date was close to the date of diagnosis of the cases since cases were interviewed on average within three months of diagnosis.

The family history of cancer was evaluated separately for mothers, fathers, brothers and sisters, and then among all first-degree relatives taken together. We analysed the risk of oral cavity cancer related to the cancer site among relatives: all sites together, head and neck (including oral cavity, pharynx, larynx, nasal cavity and sinuses) and non-head and neck cancers. Because a high number of cancers in family members were reported non-specifically as “cancer of the head and neck”, we chose to group the locations of head and neck cancers to reduce potential inaccurate reporting of cancer subsites. We nevertheless performed some analyses for family history of specific head and neck cancer sites among all first-degree relatives.

We also conducted analyses stratified by tobacco and alcohol consumption. We also performed the same analyses using a more restricted definition of the oral cavity excluding base of tongue (C01), lingual tonsils (C02.4), soft palate (C05.1) and uvula (C05.2), since these subsites are often included in the oropharynx. In addition, seven subsites (base of the tongue, mobile tongue, floor of the mouth, gums, soft palate, hard palate, and other parts of the oral cavity) were compared for family history of cancer and personal history of other medical conditions using unconditional polytomous logistic regression.

Statistical analyses were conducted using STATA software version 10.0 (StataCorp, Texas, USA).

## Results

Among the 689 cases, the most common tumour location was floor of the mouth (188 cases, 27.3%), followed by mobile tongue (162 cases, 23.5%) and base of the tongue (130 cases, 18.9%). Less frequent tumour locations were: other parts of the oral cavity (81 cases, 11.7%), soft palate (74 cases, 10.7%), gums (37 cases, 5.4%), and hard palate (17 cases, 2.5%). The analysis using the restricted definition of oral cavity involved 485 cases.

The main characteristics of cases and controls are presented in Table 1.

**Table 1:** Main characteristics of cases and controls

	**Cases**	**Controls**	p^*^
	**N *(%)***	**N *(%)***	
**Gender**			p = 0.12
Male	556 *(80.7)*	2720 *(78.1)*	
Female	133 *(19.3)*	761 *(21.9)*	
**Area of residence (“département”)**			p < 0.001
Calvados	65 *(9.4)*	458 *(13.1)*	
Doubs/Territoire de Belfort	9 *(1.3)*	134 *(3.8)*	
Hérault	71 *(10.3)*	437 *(12.5)*	
Isère	82 *(11.9)*	497 *(14.3)*	
Loire Atlantique	127 *(18.4)*	404 *(11.6)*	
Manche	68 *(9.9)*	311 *(8.9)*	
Bas Rhin	62 *(9.0)*	451 *(12.9)*	
Haut Rhin	14 *(2.0)*	118 *(3.4)*	
Somme	145 *(21.0)*	476 *(13.7)*	
Vendée	46 *(6.8)*	195 *(5.8)*	
**Age at interview**			p < 0.001
< 50	161 *(23.4)*	807 *(23.2)*	
50-59	293 *(42.5)*	995 *(28.6)*	
60-69	171 *(24.8)*	1160 *(33.3)*	
≥ 70	64 *(9.3)*	519 *(14.9)*	
**Education level**			p < 0.001
Primary or less	227 *(32.9)*	763 *(21.9)*	
Vocational secondary	294 *(42.7)*	1351 *(38.8)*	
General secondary	60 *(8.7)*	400 *(11.5)*	
University	85 *(12.3)*	901 *(25.9)*	
**Tobacco status**			p < 0.001
Never-smokers	53 *(7.7)*	1233 *(35.4)*	
Former smokers^†^	152 *(22.1)*	1436 *(41.2)*	
Current smokers	482 *(70.0)*	803 *(23.1)*	
**Quantity of tobacco (grams/day)**			p < 0.001
Never-smokers	53 *(7.7)*	1233 *(35.4)*	
1-19	275 *(39.9)*	1486 *(42.7)*	
20-39	271 *(39.3)*	600 *(17.2)*	
≥ 40	85 *(12.3)*	146 *(4.2)*	
**Duration of tobacco smoking (years)**			p < 0.001
Never-smokers	53 *(7.7)*	1233 *(35.4)*	
1-30	179 *(26.0)*	1454 *(41.8)*	
31-40	284 *(41.2)*	468 *(13.4)*	
> 40	170 *(24.7)*	313 *(7.1)*	
**Alcohol consumption (glasses/day)**			p < 0.001
Never-drinkers	38 *(5.5)*	298 *(8.6)*	
< 0.6	60 *(8.7)*	908 *(26.1)*	
0.6-2.0	74 *(10.7)*	914 *(26.2)*	
2.1-4.5	140 *(20.3)*	771 *(22.1)*	
> 4.5	360 *(52.2)*	570 *(14.8)*	
**BMI 2 years before the interview**			p < 0.001
< 18.5	28 *(4.1)*	38 *(1.1)*	
18.5-24.9	380 *(55.2)*	1367 *(39.3)*	
25-29.9	188 *(27.3)*	1367 *(39.3)*	
≥ 30	65 *(9.4)*	577 *(16.6)*	

^*^p values are derived from the Pearson’s chi-square test for categorical variables or Student’s tests for continuous variables.

^†^Former smokers were subjects who had stopped smoking for at least 2 years.

Men represented more than two-thirds of subjects in both cases and controls. Cases were younger (mean age around 57 years) than controls (mean age around 59 years) (p < 0.001).

Compared with controls, cases had a lower education level (p < 0.001), a higher consumption of tobacco (p < 0.001) and alcohol (p < 0.001), and a lower BMI two years before the interview (p < 0.001).

### Personal medical conditions

Statistical analysis showed significant positive associations between the risk of oral cavity cancer (C01-C06) and chronic bronchitis (OR 1.7, 95% CI 1.2-2.4) (Table 2). Histories of tuberculosis and candidiasis overall were associated with an increased risk of oral cavity cancer (ORs 1.6), but the results did not reach statistical significance. Among candidiasis locations, oral candidiasis was associated with an increased risk of oral cavity cancer (OR 5.0, 95% CI 2.1-12.1). Significant inverse relations were observed between the risk of oral cavity cancer and recurrent rhinitis (OR 0.6, 95% CI 0.4-0.9), nasal polyps (OR 0.3, 95% CI 0.1-0.9), and gastro-oesophageal reflux (OR 0.5, 95% CI 0.4-0.7). Herpetic lesions were not related to the risk of oral cavity cancer, regardless of the location of the herpes. The risks associated with a history of skin warts were reduced, but the results did not reach statistical significance.

**Table 2:** Risks of oral cavity cancer associated with personal medical conditions

**Personal medical conditions**	**Cases N**	**Controls N**	OR (95% CI)^*^
**Tuberculosis**			
Never	650	3228	1 (reference)
Ever	29	136	1.6 (0.9-2.5)
**Chronic bronchitis**			
Never	571	3032	1 (reference)
Ever	105	343	1.7 (1.2-2.4)
**Asthma**			
Never	632	3090	1 (reference)
Ever	48	284	1.1 (0.7-1.7)
**Recurrent rhinitis**			
Never	623	2843	1 (reference)
Ever	53	519	0.6 (0.4-0.9)
**Recurrent nose bleeds**			
Never	632	3043	1 (reference)
Ever	45	327	0.9 (0.6-1.4)
**Nasal polyps**			
Never	672	3251	1 (reference)
Ever	5	113	0.3 (0.1-0.9)
**Recurrent sinusitis**			
Never	590	2796	1 (reference)
Ever	88	578	0.7 (0.5-1.0)
**Gastro-oesophageal reflux**			
Never	591	2612	1 (reference)
Ever	85	753	0.5 (0.4-0.7)
**Herpetic lesions**			
Never	625	2964	1 (reference)
Ever^‡^	49	400	0.9 (0.6-1.4)
Lip	41	347	0.8 (0.5-1.2)
Genitals	4	34	1.2 (0.4-3.9)
**Candidiasis**			
Never	645	3224	1 (reference)
Ever^‡^	28	136	1.6 (0.9-2.8)
Oral cavity	14	80	5.0 (2.1-12.1)
Genitals	6	47	1.0 (0.3-2.7)
**Skin warts**			
Never	461	1909	1 (reference)
Ever^§^	215	1458	0.8 (0.6-1.1)
Hands	170	1101	0.8 (0.6-1.0)
Feet	39	327	0.7 (0.5-1.2)
Head and neck	8	111	0.5 (0.2-1.3)

^*^OR adjusted for age, gender, area of residence, education level, tobacco smoking (duration, quantity and status), alcohol drinking (quantity) and BMI two years before the interview.

^‡^Location of herpetic lesions and candidiasis not reported by 24 subjects and 17 subjects, respectively.

^§^Multiple locations reported by 83 subjects.

Because high prevalence of oropharyngeal candidiasis has been described in subjects with head and neck cancer undergoing radio/chemotherapy [33,34], we also conducted analysis after excluding subjects declaring candidiasis at the time of interview or at the time of diagnosis of another cancer; the association between oral cavity cancer risk and oral candidiasis remained practically unchanged (OR 6.0, 95% CI 2.2-16.4). Oral candidiasis may also constitute an early manifestation of the cancerous disease. When we excluded all subjects reporting a history of oral candidiasis near the current cancer (up to two years before the cancer diagnosis), the association between oral cavity cancer and oral candidiasis remained significant (OR 3.7, CI 95% 1.3-10.1).

We calculated the ORs for oral candidiasis in strata of tobacco and alcohol consumption. The OR was slightly higher in ever smokers (OR 5.6, 95% CI 2.0-15.2) than in never smokers (OR 4.3, 95% CI 0.5-41.7), but the interaction between tobacco smoking and oral candidiasis was not significant (*p*-value for interaction = 0.14). Oral candidiasis was associated with an elevated risk of oral cavity cancer in drinkers of more than 2 glasses/day (OR 3.9, 95% CI 1.0-14.8) but not in drinkers of 2 glasses/day or less (OR 1.1, 95% CI 0.2-23.8), although the ORs were not statistically different (*p*-value for interaction = 0.50).

When the analysis was restricted to medical conditions that the subjects reported as diagnosed by a doctor, similar results were observed, except for the association between bronchitis and oral cavity cancer which became weaker and non-significant (OR 1.2, 95% CI 0.9-1.6) (data not shown).

### Family history of cancer

The associations between family history of cancer and risk of oral cavity cancer are presented in Table 3.

**Table 3:** Risks of oral cavity cancer associated with family history of cancer among first-degree relatives

	**Cases N**	**Controls N**	**OR (95% CI)**^**†, ‡**^
**Family history of cancer (father)**			
**All type of cancer**			
Never	430	2503	1 (reference)
Ever	185	796	^†^1.3 (0.9-1.6)
**Head and neck cancer**			
Never	576	3200	1 (reference)
Ever	39	99	^†^1.5 (0.9-2.4)
**Family history of cancer (mother)**			
**All type of cancer**			
Never	543	2784	1 (reference)
Ever	97	603	^†^0.9 (0.6-1.2)
**Head and neck cancer**			
Never	633	3378	1 (reference)
Ever	7	9	^†^5.2 (1.2-23.9)
**Family history of cancer (brothers)**			
**All type of cancer**			
Never	229	1218	1 (reference)
Ever	77	288	^‡^1.4 (0.9-2.1)
**Head and neck cancer**			
Never	286	1467	1 (reference)
Ever	20	39	^‡^2.6 (1.2-5.8)
**Family history of cancer (sisters)**			
**All type of cancer**			
Never	190	1236	1 (reference)
Ever	72	309	^‡^1.4 (0.9-2.1)
**Head and neck cancer**			
Never	251	1517	1 (reference)
Ever	11	28	^‡^1.7 (0.6-4.2)
**Family history of cancer (any first-degree relative)**			
**All type of cancer**			
Never	250	1567	1 (reference)
Ever	333	1595	^†^1.2 (0.9-1.5)
1-2	329	1552	1.0 (0.8-1.3)
≥3	4	43	1.5 (1.0-2.1)
**Head and neck cancer**			
Never	462	2784	1 (reference)
Ever	68	157	^†^1.9 (1.2-2.8)
1-2	64	150	1.7 (1.1-2.6)
≥3	4	7	7.2 (1.3-40.1)

^†^OR adjusted for age, gender, area of residence, education level, tobacco smoking (duration, quantity and status), alcohol drinking (quantity) and BMI two years before the interview.

^‡^OR adjusted, in addition to the variables of the first model, for the number of brothers, sisters or siblings, respectively.

History of cancer in general, and of head and neck cancer in particular, among fathers, were associated with a slightly elevated risk of oral cavity cancer, but the results were not statistically significant (OR 1.3, 95% CI 0.9-1.6, and 1.5, 95% CI 0.9-2.4, respectively).

History of head and neck cancer among mothers was significantly associated with an elevated risk of oral cavity cancer (OR 5.2, 95% CI 1.2-23.9). This OR was higher than that observed for fathers. When cancer history among siblings was analysed, after adjustment for the number of sisters and brothers, we observed a significant association between the risk of oral cavity cancer and history of cancer of any type (OR 1.4, 95% CI 1.1-1.9). History of head and neck cancer among siblings was associated with an increased risk of oral cavity cancer (OR 2.3, 95% CI 1.2-4.2). Analysis by type of sibling showed a significantly increased risk only among subjects having brothers with a history of head and neck cancer (OR 2.6, 95% CI 1.2-5.8); history of head and neck cancer among sisters was not significantly associated with an increased risk of oral cavity cancer (OR 1.7, 95% CI 0.6-4.2).

When we analysed the relationship between cancer history among all first-degree relatives and oral cavity cancer risk, we observed significant association for history of head and neck cancer (OR 1.9, 95% CI 1.2-2.8). A family history of any type of cancer slightly increased the risk of oral cavity cancer, but the results were not statistically significant (OR 1.2, 95% CI 0.9-1.5). The risk associated with first-degree relatives’ history of cancer (any type and head and neck) increased with the number of affected relatives.

Analysis by type of head and neck cancer in first-degree relatives showed significantly increased risks of oral cavity cancer in subjects with family history of oral cavity cancer (OR 3.5, 95% CI 1.1-11.2) and of “head and neck cancer” (not specified) (OR 1.8, 95% CI 1.1-2.9). A family history of pharyngeal, laryngeal, and sinonasal cancer was associated with non-significantly elevated risks of oral cavity cancer (OR 4.6, 95% CI 0.5-44.8 for history of pharyngeal cancer; 1.6, 95% CI 0.6-4.4 for history of laryngeal cancer; and 1.7, 95% CI 0.3-8.8 for history of sinonasal cancer). However, few subjects reported a specific location of head and neck cancer in first degree relatives (26 oral cavity, 5 pharynx, 35 larynx, and 11 nasal cavity/sinuses cancer).

Analysis stratified by gender of first-degree relatives showed that history of head and neck cancer among female relatives (mothers and sisters) was not significantly associated with the risk of oral cavity cancer (OR 2.3, 95% CI 0.9-5.4), although the result was borderline significant. Conversely, history of head and neck cancer in male relatives (fathers and brothers) was significantly associated with the risk of oral cavity cancer (OR 1.9, 95% CI 1.2-3.3). However, these ORs did not differ significantly (*p*-value of test of comparison of ORs = 0.91).

We found a stronger association between the risk of oral cavity cancer and family history of head and neck cancer in subjects aged 45 or more (OR 2.3, 95% CI 1.5-3.4) compared to subjects aged less than 45 (OR 1.3, 95% CI 0.3-6.7), although the ORs were not statistically different (*p*-value for interaction = 0.46).

Analysis by cancer site among first-degree relatives (Table 4) showed elevated ORs among subjects having a family history of lung, oesophagus, cervix and corpus uteri, brain and nervous system cancer, but the results were not statistically significant (OR 1.4, 95% CI 0.9-1.9; 1.5, 95% CI 0.7-3.3; 1.7, 95% CI 0.9-3.1; 2.0, 95% CI 0.9-4.8 respectively).

**Table 4:** Odds ratios for oral cavity cancer risk related to family history of selected cancers in first-degree relatives

	**Subjects with family history (any first-degree relative)**		
**Cancer site**	**Cases**	**Controls**	**OR (95% CI)**^**†**^
Lung	71	248	1.4 (0.9-1.9)
Oesophagus	12	51	1.5 (0.7-3.3)
Stomach	14	89	0.9 (0.-1.8)
Intestines	37	274	0.8 (0.5-1.3)
Liver	18	95	1.1 (0.6-2.1)
Pancreas	8	55	0.7 (0.3-1.8)
Thyroid gland	3	15	0.8 (0.2-3.2)
Bones and cartilages	10	43	1.1 (0.4-2.6)
Skin	5	65	0.3 (0.1-1.1)
Prostate	27	172	1.2 (0.7-1.9)
Other male genital tract	2	11	1.2 (0.2-7.4)
Breast	56	312	1.0 (0.7-1.5)
Cervix and corpus uteri	21	108	1.7 (0.9-3.1)
Other female genital tract	3	28	0.4 (0.2-2.2)
Bladder	4	39	0.7 (0.3-2.2)
Other urinary tract	8	55	0.6 (0.2-1.7)
Brain and nervous system	16	49	2.0 (0.9-4.8)
Lymphoma, leukaemia	18	122	1.1 (0.6-2.1)
Other and unspecified sites	9	40	1.0 (0.3-2.8)

^†^ORs adjusted for age, gender, area of residence, education level, tobacco smoking (duration, quantity and status), alcohol drinking (quantity) and BMI two years before the interview.

When we stratified by tobacco smoking and/or alcohol drinking (Table 5), significantly increased risks of oral cavity cancer related to family history of any type of cancer were observed only in smokers and/or moderate to heavy drinkers. Significantly elevated risks of oral cavity cancer associated with family history of head and neck cancer were seen for both never and ever smokers and for light and moderate to heavy drinkers. However, the increase in risk was small and not significant for never smokers who were also light drinkers.

**Table 5:** Risks of oral cavity cancer related to family history of cancer in first-degree relatives stratified by tobacco smoking and alcohol drinking

	**Any first-degree relative**					
	**History of cancer (any site)**			**History of head and neck cancer**		
	^**‡**^**No**	**Yes**	**OR (95% CI)**^**†**^	^**§**^**No**	**Yes**	**OR (95% CI)**^**†**^
	**Cases/controls**	**Cases/controls**		**Cases/controls**	**Cases/controls**	
**Smoking**						
Never	19/563	27/559	1.1 (0.6-2.1)	35/994	6/51	2.4 (1.0-7.0)
Ever	231/1002	306/1030	1.3 (1.1-1.5)	427/1785	58/104	2.7 (1.8-3.4)
**Drinking**						
≤2 glasses/day	75/961	75/954	0.9 (0.6-1.4)	123/1704	11/88	1.9 (1.0-3.5)
>2 glasses/day	167/597	254/631	1.4 (1.0-1.8)	329/1066	52/67	2.6 (1.2-3.3)
**Smoking & drinking**						
NS & D ≤2 glasses/day	16/412	17/425	0.9 (0.4-1.9)	30/740	1/39	1.2 (0.3-4.7)
ES & D ≤2 glasses/day	59/548	58/526	0.9 (0.6-1.4)	93/962	10/47	1.6 (1.0-3.9)
NS & D >2 glasses/day	3/147	8/129	2.7 (0.6-11.6)	5/248	4/10	18.8 (2.9-119.8)
ES & D >2 glasses/day	164/449	246/499	1.4 (1.0-1.8)	324/815	48/57	2.1 (1.3-2.9)

^†^OR adjusted for age, gender, area of residence, education level, tobacco smoking (duration, quantity and status), alcohol drinking (quantity) and BMI two years before the interview.

NS = never smoking; ES = ever smoking; D = drinking.

^‡^Reference category: no history of cancer (any site) in first-degree relatives.

^§^Reference category: no history of head and neck cancer in first-degree relatives.

### Analyses restricted to intraoral cavity

When the analyses were limited to intraoral cavity (C02.0-C02.3, C02.8, C02.9, C03, C04, C05.0, C05.8, C05.9, C06), the results were similar to that observed for oral cavity globally (C01-C06). Thus, family history of UADT cancer among first-degree relatives was associated with an OR of 1.7 (95% CI 1.1-2.7), personal history of oral candidiasis with an OR of 4.9 (95% CI 1.8-13.3), gastro-oesophageal reflux with an OR of 0.6 (95% CI 0.4-0.8), recurrent rhinitis with an OR of 0.6 (95% CI 0.4-0.9), and nasal polyps with an OR of 0.3 (95% CI 0.1-0.9).

### Analyses by subsite

We assessed the risk of oral cavity cancer by anatomical site of the oral cavity (base of tongue, mobile tongue, gum, floor of mouth, hard and soft palate, and other parts of oral cavity) for personal medical conditions and for family history of cancer using a polytomous regression. We did not find any difference between subsites for any variable of interest (tests of comparison of odds ratios non-significant) (data not shown).

## Discussion

To our knowledge, the ICARE study is the first population-based case–control study in France and one of the largest in the world which investigates the role of risk factors other than tobacco and alcohol consumption in the occurrence of oral cavity cancer. Strengths of this study include large sample size allowing us to perform analyses by subsite, and detailed data about family history of cancer and personal medical history.

The ICARE study was conducted in collaboration with the cancer registries, allowing us to recruit cancer cases in all healthcare establishments in the selected areas. The control group was population-based and common for both pathologies (lung and UADT cancers), which explains the significantly different distribution of age and area of residence between oral cavity cancer cases and controls. However, the large number of subjects in each category allowed for satisfactory adjustment for these variables.

The results of the epidemiological studies are contrasted concerning the role of candidiasis in the occurrence of oral cavity cancer, Thus, history of oral candidiasis was associated with an increased risk of oral cavity and oropharyngeal cancer in one study [11], with a reduced risk of oral cavity cancer in another study [7], whereas other authors found no association [6]. Our results have shown that personal history of oral candidiasis was associated with an elevated risk of oral cavity cancer. The increase in cancer risk with oral candidiasis may be explained by the production of endogenous nitrosamines by *Candida albicans*[35]. These nitrosamines act on the normal epithelium leading to oral dysplasia and further development of oral carcinoma. Nevertheless, some authors suggested that *Candida albicans* have only an indirect role and that the possibility of their involvement exist in conjunction with other etiological factors such as tobacco smoking [36]. In our study, the risk of oral cavity cancer associated with history of candidiasis was slightly higher in smokers than in never smokers, but the ORs were not significantly different. Other studies showed that *Candida albicans* may metabolize ethanol into its carcinogenic metabolite, acetaldehyde and, accordingly, candidiasis may be associated with elevated acetaldehyde levels in the oral cavity [37,38]. Consistent with this mechanism, we found a significantly elevated risk of oral cavity cancer associated with history of candidiasis in moderate to heavy drinkers but not in light drinkers. However, the interaction of oral candidiasis with alcohol drinking was not statistically significant. Also, chronic infections, specifically chronic hyperplastic candidiasis, may trigger cell proliferation, inhibit apoptosis, interfere with cellular signalling mechanisms and up-regulate tumour promoters [39,40]. In our study only 14 cases and 80 controls reported prior candidiasis and the results should be confirmed by other studies, especially with medical conditions validated by a doctor.

In agreement with previous studies [7,8,11], we did not find a significant association between the risk of oral cavity cancer and history of herpetic infection. Conversely, only one case–control study [10] found an increased risk of oral cavity and oropharynx cancer associated with this infection and two case–control studies [6,9] found a decreased risk.

Cutaneous warts are caused by different types of HPV, notably 2, 4, 7 and 57, whereas genital warts are caused mostly by HPV types 6 and 11 [41]. Three studies [6,8,11], like ours, did not find any association between history of warts (any location) and the risk of oral cavity cancer, whereas one study found a reduced risk of UADT cancer associated with feet, genital and head and neck warts [7].

We found an inverse association between the oral cavity cancer risk and history of rhinitis and nasal polyps. These pathologies often have an allergic origin, and several studies found a decreased risk of head and neck cancer associated with a history of allergies [42-45]. The inverse association between allergies and cancer may be explained by an overactive immune function in allergic subjects that effectively detects and eradicates malignant cells, toxins or pathogens from the body [46,47]. However, we did not find any association with the history of asthma, another allergies-related condition.

We found also an inverse association between oral cavity cancer risk and history of gastro-oesophageal reflux but we cannot point to any specific mechanism. The possibility that this result is due to the chance may not be ruled out. Unlike our results, a recent case–control study [7] did not find any association between oral cavity cancer risk and gastro-oesophageal reflux.

After controlling for main confounding factors, we observed a higher risk of oral cavity cancer among subjects having first-degree relatives with head and neck cancer history, compared to subjects without such a family history. The risk increased with the number of affected relatives. On the other hand we did not find a significant relationship between the risk of oral cavity cancer and family history of non-head and neck cancers. Several studies [20,22,23,26] reported similar results.

Early age of onset may be a feature of hereditary forms of cancer. Higher family risks for many cancers were found when the cancer subjects were diagnosed at an early age [48,49]. Concerning the association between the risk of oral cancer and family history of head and neck cancer, no clear pattern emerges from epidemiological studies: some of them found a stronger association in younger subjects compared to older subjects [20,21,23], others found a contrary result [22,26], but the differences in risk with age of onset were never significant. Similarly, in our study the interaction of family history of head and neck cancer with age was not significant, although the OR was somewhat higher in older subjects.

Familial clustering of cancer cases could be explained by genetic polymorphism in genes involved in the metabolism of tobacco and alcohol carcinogens and DNA repair [12-19], but may also reflect a tendency of relatives to have similar behaviour concerning alcohol and tobacco.

In our study, associations between oral cavity cancer risk and family history of cancer were observed among smokers and/or drinkers of >2 glasses/day only. Conversely, an increased risk of oral cavity cancer associated with a family history of head and neck cancer was also observed in non-smokers and light drinkers, but the risk increased with the exposure. Our results are similar to those of other studies on oral/pharyngeal or head and neck cancer [20,23,26].

The differential ability of subjects to metabolize carcinogens when exposure to tobacco and/or alcohol occurs may explain the higher risk of oral cavity cancer observed in our study among smokers and drinkers having a family history of UADT cancer. Nevertheless, we did not observe an increased risk of oral cavity cancer in subjects having a family history of other cancers related to smoking and/or alcohol drinking (e.g. lung, oesophagus, liver, pancreas), suggesting that other genetic factors might explain these findings.

When the analyses were limited to intraoral cavity (C02.0-C02.3, C02.8, C02.9, C03, C04, C05.0, C05.8, C05.9, C06), excluding the sites usually attached to oropharynx (C01, C02.4, C05.1, C05.2), the results were similar to that observed for oral cavity C01-C06. We did not find any difference between subsites base of the tongue, mobile tongue, gums, floor of the mouth, soft palate, hard palate, and other parts of the mouth for any variable of interest.

Some limitations of our study can be discussed. The subjects self-reported their own medical history and family history of cancer. Thereby, recall bias could not be ruled out and it is possible that the cases had a higher motivation to recall their personal and family medical history than the controls. Nevertheless, two studies have shown that subjects in case–control studies are able to report accurately family history of common types of cancer among first-degree relatives, with little observable recall bias [50,51]. In addition, family history of cancer sites other than head and neck was not associated with an increased risk of oral cavity cancer in this study, suggesting that no major recall bias concerning cancer in general has affected our results.

With regards to oral candidiasis, a possible explanation would be that cases with oral cancer are more prone to recall previous oral lesions than controls. However, no association was found with labial herpes, another oral condition, suggesting that differential recall between cases and controls is unlikely to explain our results. Moreover, when we limited the medical conditions to those reportedly diagnosed by a doctor, the results were similar. Medical treatments were also collected and those reported for candidiasis were consistent with this pathology. So, we think that misclassification of candidiasis is unlikely to explain our results.

Information about other known risk factors for oral cavity cancer such as diet, human papilloma virus (HPV) or dental health was not collected, and residual confounding cannot be excluded. Nevertheless, no association between diet, HPV infection or dental health and family history of cancer has ever been shown in the literature. Also, the possibility of residual confounding for the main risk factors may not be ruled out.

We have no way to assess whether cases included in our study differed according to past medical conditions and family history of cancer from cases that could not be included. Nevertheless, the distribution of included cases by age, gender and cancer subsite was very similar to that of all oral cancer cases diagnosed in France [52], suggesting that no major selection bias occurred. We excluded from analysis all subjects with shortened questionnaires because information on medical conditions and family history of cancer was not available. However, these subjects were comparable in age, gender, and tobacco and alcohol consumption to subjects with complete questionnaires.

## Conclusion

This study showed that family history of head and neck cancer is related to an increased risk of oral cavity cancer, and suggested an association with personal history of oral candidiasis. From a public health point of view, these factors should be taken into account to target prevention efforts and screening.

## Competing interest

The authors declare that they have no conflict of interest.

## Authors’ contributions

DL and LR conceived and designed the current study and drafted the manuscript; LR and DC analyzed the data; DL and IS are the principal investigators of ICARE, conceived the study, designed the questionnaire, and coordinated the original collection of the data. AS, DC, SC, MS, AVG and BT contributed to data collection and quality control; SPB, FG, GM, MC contributed to the statistical analysis. All authors participated to data interpretation and critical revision of the manuscript. All authors read and approved the final manuscript.

## Pre-publication history

The pre-publication history for this paper can be accessed here:

http://www.biomedcentral.com/1471-2407/13/560/prepub
